# Two-Step Cell Death Induction by the New 2-Arachidonoyl Glycerol Analog and Its Modulation by Lysophosphatidylinositol in Human Breast Cancer Cells

**DOI:** 10.3390/ijms26020820

**Published:** 2025-01-19

**Authors:** Mikhail G. Akimov, Natalia M. Gretskaya, Evgenia I. Gorbacheva, Nisreen Khadour, Galina D. Sherstyanykh, Vladimir V. Bezuglov

**Affiliations:** 1 Shemyakin-Ovchinnikov Institute of Bioorganic Chemistry, Russian Academy of Sciences, Miklukho-Maklaya 16/10, 117997 Moscow, Russia; natalia.gretskaya@gmail.com (N.M.G.); gorevig2000@gmail.com (E.I.G.); nisreenkhadour75@gmail.com (N.K.); galya24may@gmail.com (G.D.S.); vvbez@ibch.ru (V.V.B.); 2 Moscow Center for Advanced Studies, Kulakova Str. 20, 123592 Moscow, Russia

**Keywords:** CB1, CB2, TRPV1, LPI, 2-arachidonoylglycerol, COX-2, breast cancer

## Abstract

2-arachnadoyl glycerol (2-AG) is one of the most common endocannabinoid molecules with anti-proliferative, cytotoxic, and pro-proliferative effects on different types of tumors. Typically, it induces cell death via cannabinoid receptor 1/2 (CB1/CB2)-linked ceramide production. In breast cancer, ceramide is counterbalanced by the sphingosine-1-phosphate, and thus the mechanisms of 2-AG influence on proliferation are poorly understood. We evaluated the mechanism of the anti-proliferative action by 2-AG and the influence of lysophaosphatidylinositol (LPI) on it in six human breast cancer cell lines of different tumor degree (MCF-10A, MCF-7, BT-474, BT-20, SK-BR-3, and MDA-MB-231) using resazurin test, inhibitor, blocker, and anti-oxidant analysis, and siRNA interference. To avoid acyl migration in 2-AG, we replaced it with the analog 2-arachidonoyl-1,3-difluoropropanol (2-ADFP) newly synthesized by us. Using a molecular docking approach, we showed that at the CB2 receptor, 2-ADFP and 2-AG were very close to each other. However, 2-ADFP demonstrated a stronger affinity towards CB1 in the antagonist-bound conformation. 2-ADFP was anti-proliferative in all the cell lines tested. The toxicity of 2-ADFP was enhanced by LPI. 2-ADFP activity was reduced or prevented by the CB2 and vanilloid receptor 1 (TRPV1) blockers, inositol triphosphate receptor, CREB, and cyclooxygenase 2 inhibitor, and by anti-oxidant addition. Together with the literature data, these results indicate CB2- and TRPV1-dependent COX-2 induction with concomitant cell death induction by the oxidized molecule’s metabolites.

## 1. Introduction

2-arachidonoyl glycerol (2-AG) is one of the most common endocannabinoid molecules and is usually produced on demand by mesenchymal stromal cells in the bone marrow [1]. The degradation of 2-AG is predominantly mediated by monoacylglycerol lipase (MAGL) or α/β-hydrolase domain containing 6 (ABHD6) and 12 (ABHD12) [2]. The receptors activated by 2-AG are represented by the G_i/o_ protein-bound cannabinoid receptors CB1 and CB2 [3,4]. The affinity for CB2 in 2-AG is greater than for CB1. The vanilloid receptor TRPV1 is an ion channel that can also be activated by 2-AG at concentrations of tens to hundreds of micromoles [5]. In the context of cancer cells, 2-AG has anti-proliferative, cytotoxic, and pro-proliferative effects on different types of tumors, and is also able to stimulate or, conversely, inhibit cell migration.

Cell migration is inhibited by 2-AG through the activation of the CB1 receptor, followed by the inhibition of adenylate cyclase activity. This can lead to the disruption of the signal transmission pathway to protein kinase A, which regulates cell metastasis. This effect of 2-AG was manifested in micromolar concentrations [6]. The activation of cell migration occurs through the CB2 receptor, resulting in the phosphorylation of ERK1/2, which leads to an increase in the cell mobility of B-cell leukemia. In this case, micromolar concentrations (microns) are also needed to activate the signaling pathway [7,8].

The anti-proliferative effect of 2-AG is typically realized through the CB1 receptor, which is known to inhibit breast cancer cell proliferation [9]. This way, 2-AG reduces viability and induces apoptosis in DU-145 [10], MCF-7 [11], and in part in the Hodgkin’s lymphoma cells [12]. However, the cytotoxic effect on the tumor cells of the larynx HNSCC and oral cavity SNU-1066 is associated with the activation of the CB2 receptor [13]. In addition, the CB2 agonist induced cytotoxicity and apoptosis in human colorectal cancer cells (HT-29) [14], and the selective CB2 agonist JWH-133 induced a considerable regression of malignant tumors generated by the inoculation of C6 glioma cells [15].

The mechanisms by which the CB1 receptor activation reduces proliferation and induces apoptosis are rather well known and it typically occurs via ceramide biosynthesis stimulation [16]. CB2 activation may also lead to ceramide biosynthesis as a part of the apoptosis induction [17]. Ceramide, in part, is able to induce endoplasmic reticulum stress and the subsequent apoptosis [18]. However, in the breast cancer setting, ceramide biosynthesis induction may cause a pro-survival effect due to the high rate of conversion to sphingosine-1-phosphate [19]. In addition, CB2 activation in breast cancer cells was shown to modulate viability in a calcium-dependent fashion [20]. In addition, a prolonged treatment by the CB2 agonists could have a pro-survival effect on cancer cells [21]. These data suggest the existence of multiple mechanisms by which 2-AG could induce cell death, especially in the breast cancer setting, and their elucidation could be important for the understanding of the role of this substance in cancer development.

In addition to autonomous effects, the 2-AG CB2 receptor is involved in interreceptor interaction. As such, the heterodimers of CB2 with the chemokine receptor CXCR4 [22], adenosine receptor (A2aR) [23], and HER2 [24] were described. Of special interest are the heterodimers with other cannabinoid receptors, as they could lead to an unexpected interaction between various endocannabinoids. As such, in the CB1-CB2 complex, CB2 blocks the effects mediated by the CB1 receptor [25]. GPR55-CB2 heterodimers were also identified [26], pointing to the possibility of an interaction between the pro-cancer LPI signaling axis [27] and the 2-AG function.

The use of 2-AG in biological experiments is associated with several difficulties since its molecule can undergo oxidation by the enzymes of the arachidonic acid cascade [28], as well as the hydrolysis of the ester bond and acyl migration. In the context of biological experiments, both the oxidation and hydrolysis of the ester group will be rather slow processes under physiological conditions and will only be significant in experiments lasting more than 4 h [29]. 2-Monoglycerides are subject to acyl migration because the 1(3)-isomer is thermodynamically more stable than the 2-isomer. The reaction continues until equilibrium is reached, and the equilibrium mixture contains no more than 5–10% of the 2-isomer [29]. Therefore, there is no certainty that the experimenter acts on the object with 2-AG, and not a mixture of 1,3-isomers. To avoid acyl migration, we synthesized an analog of 2-AG in which the hydroxyl groups were replaced by a fluorine atom. The electronic properties and relatively small size of the fluorine atom give it considerable versatility as a biosphere, and it can act as a functional mimetic of hydroxyl and other functional groups [30]. We used this approach to synthesize the fluorinated analog of 2-AG.

In this paper, we evaluated the mechanism of cell death indication by 2-AG in six human breast cancer cell lines of different tumor degrees, and the influence of LPI on it. 2-AG induced cell death via the CB2- and TRPV1-dependent COX-2 induction and oxidized metabolite production. The activity was enhanced by LPI.

## 2. Results

### 2.1. Cell Lines

The topic of this research was the direction and mechanism of 2-AG and LPI combined activity. As with the case of anandamide in our previous study [31], we assumed that at least the direction and magnitude of this interaction could depend on the grade of a tumor, as the cells progressively accumulate mutations and their response to various stimuli change. To address this possibility, a panel of human breast cancer cell lines with different properties was used (Table 1). These lines were chosen to represent all of the major breast cancer subtypes [32,33].

### 2.2. Synthesis and Characterization of the 2-AG Analog 2-Arachidonoyl-1,3-Difluoropropanol and Its Activity for the DU 145 Cell Line

**Figure 1:**
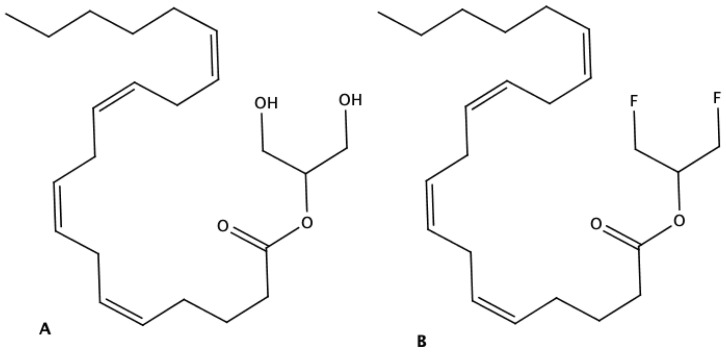
The structure of 2-AG (**A**) and 2-ADFP (**B**).

Given the low stability and propensity to acyl migration of 2-AG, its analog 2-arachidonoyl-1,3-difluoropropanol (2-ADFP) with hydroxyl groups replaced by fluorine was synthesized and used in all the experiments (Figure 1). The synthesis of 2-ADFP was performed by the esterification of arachidonic acid with 1,3-difluoropropanol via fluoro anhydride method (see Section 4).

The further logic of the research was as follows: First, we verified that the docking affinities of 2-AG and 2-ADFP for the CB2 receptor are close to each other. Then, using a cell line with a known CB2 receptor expression and siRNA for this receptor, we demonstrated that 2-ADFP affects cell proliferation and that these effects are reduced or abolished after CB2 knockdown. After that, we compared the activity of 2-ADFP in different breast cancer cell lines alone and in combination with LPI, and checked the involved receptor.

**Figure 2:**
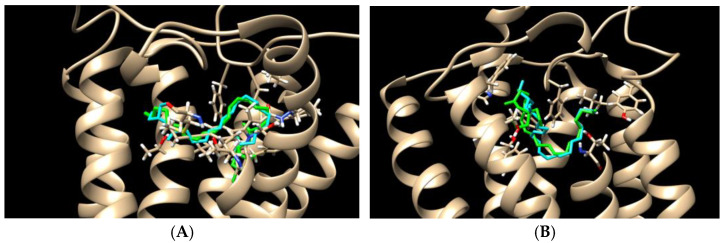
Molecular docking of 2-ADFP in the active site of the CB1 (**A**) and CB2 (**B**) receptors. Cyan, 2-ADPG; green, 2-AG.

To validate the molecular target of 2-ADFP, we used a molecular docking approach and a cellular model. First, the affinity of the molecule to the active sites of the CB2 and CB1 receptors was estimated in the molecular docking experiments in comparison to the unmodified 2-AG (Table 2, Figure 2). The affinity scores of the substances were very close to each other; however, 2-ADFP demonstrated a stronger affinity towards CB1 in the antagonist-bound conformation.

### 2.3. 2-ADFP Activity for the DU 145 Cell Line and CB2 as Its Molecular Target

**Figure 3:**
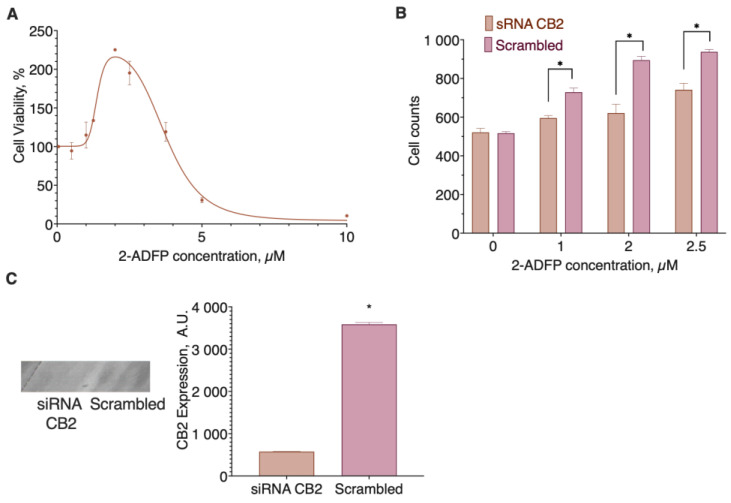
CB2 receptor role in the DU145 cell line response to the 2-ADFP action. (**A**) The influence of 2-ADPG concentration on cell viability; (**B**) the effect of the CB2 receptor knockdown on the pro-proliferative 2-ADFP activity; (**C**) the siRNA knockdown of the CB2 receptor in the DU 145 cell line. Incubation time 72 h, resazurin test, mean ± standard error (N = 4 experiments). *, a statistically significant difference from the cells transfected with the scrambled siRNA, *p* ≤ 0.05, ANOVA with Holm–Sidak post-test (**B**) and Student’s *t*-test (**C**).

Next, we tested 2-ADFP activity on the DU 145 cell line, for which a substantial expression of the CB2 receptor, the primary target of 2-AG, is known [39]. As a control, we silenced the receptor using siRNA (Figure 3).

2-ADFP stimulated cell proliferation in the concentration range from 1 to 3 µM (EC_50_ 1.37 (1.023 to 1.717) µM) (Figure 3A), and this activity was partially abolished after the CB2 receptor knockdown (Figure 3B,C), thus confirming the ability of the substance to interact with the receptor. The higher concentrations of the substance were cytotoxic.

### 2.4. Individual Effects of 2-ADFP on the Breast Cancer Cell Lines

The first stage of the research was to determine the individual activity of 2-ADFP for the chosen cell lines. As far as 2-AG is known to affect cell proliferation [40,41], and LPI can act as a proliferation stimulator, the incubation time was chosen to be 72 h. Cell viability was determined using the resazurin test; cell death was confirmed using microscopy.

We did not explicitly test LPI activity, as it was already described in our previous study [31].

**Figure 4:**
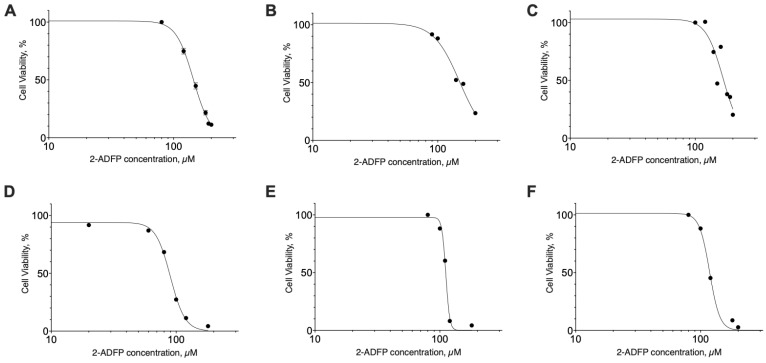
The effect of 2-ADFP on breast cancer cell line proliferation. Incubation time 72 h, resazurin test, mean ± standard error (*N* = 4 experiments). (**A**) MCF-10A, (**B**) MCF-7, (**C**) BT-474, (**D**) SK-BR-3, (**E**) BT-20, and (**F**) MDA-MB-231.

In the range from 1 to 50 µM, 2-ADFP did not affect the proliferation of the breast cancer cell lines. However, in the concentration range from 50 to 150 µM, the substance was cytotoxic with EC_50_ in the range 89–167 µM (Figure 4, Table 3). For the MCF-10A, MCF-7, and BT-474, the highest concentration of the substance (200 µM) did not kill 100% of the cells, while for the SK-BR-3, BT-20, and MDA-MB-231, all the cells died at the high substance concentrations.

### 2.5. The Effect of 2-ADFP-LPI Combinations on the Viability of the Cell Lines

We next tested the ability of LPI to change the activity of 2-ADFP when added together. We used a 2-ADFP concentration close to its EC_50_ value and several LPI concentrations to account for the possible activity changes in different concentration ranges. In this experiment series, we also used a 72 h incubation with the substances to detect possible proliferation changes and the resazurin test to evaluate cell viability.

**Figure 5:**
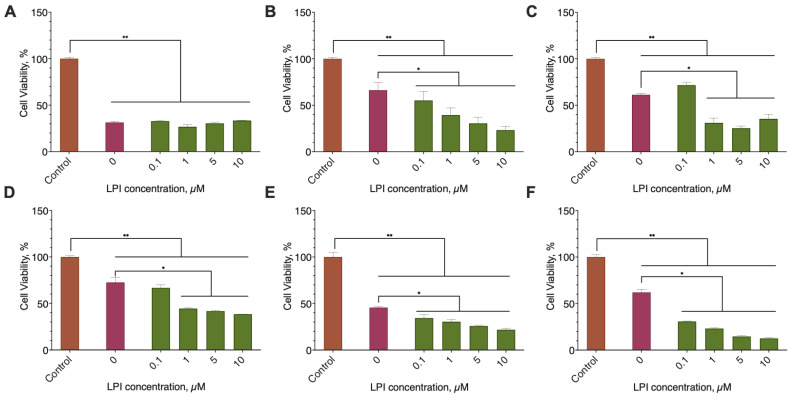
The effect of 2-ADFP combination with LPI on breast cancer cell line viability. Incubation time 72 h, resazurin test, mean ± standard error (*N* = 4 experiments). (**A**) MCF-10A, (**B**) MCF-7, (**C**) BT-474, (**D**) SK-BR-3, (**E**) BT-20, and (**F**) MDA-MB-231. *, a statistically significant difference from 2-ADFP alone, *p* ≤ 0.05, ANOVA with Holm–Sidak post-test; **, a statistically significant difference from the non-treated control, *p* ≤ 0.05, ANOVA with Holm–Sidak post-test.

In the case of the MCF-10A cell line, LPI did not affect 2-ADFP activity, while in the case of all other cell lines, the combined cytotoxicity was higher than the one of the 2-ADFP alone (Figure 5).

### 2.6. Receptor Participation in the Individual and Combined Substance Effects

The next question of our research was the mechanism of the LPI effect on 2-ADFP activity. Our hypothesis was that 2-ADFP-LPI interaction could proceed through one of the known cannabinoid receptors, as some of them (CB1 and CB2) are targets for 2-AG [42], others are targets for LPI (GPR18 and GPR55) [43], and at least for some of them, a formation of activity-changing heterodimers was described [44,45,46].

The expression of the core cannabinoid receptors (CB1, CB2, and GPR55) in the model cell lines was estimated in our previous research. MCF-7, MDA-MB-231, SK-BR-3, BT-474, and BT-20 expressed all three of them, although to a different extent. In MCF-10A, the expression of CB2 was not observed, and in MCF-7, GPR55 expression was negligible [31].

We added a selective blocker for each of those receptors both to 2-ADFP alone and to the 2-ADFP-LPI combination to check for the importance of the appropriate receptor, and evaluated the proliferation change in the resazurin test after 72 h of incubation with the cells. The following blockers and concentrations were used: CB1, SR 141716A (100 nM); CB2, SR 144528 (100 nM); GPR55, ML-193 (2 µM); GPR18, PSB CB5 (3 µM); and TRPV1, capsazepine (5 µM).

**Figure 6:**
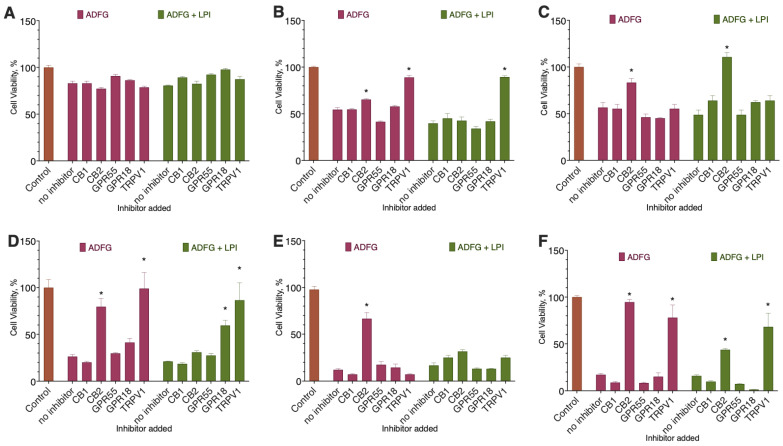
The effect of receptor blockers on the effect of 2-ADFP (EC_50_-EC_70_) combination with LPI (5 µM) on breast cancer cell line viability. The following substances and concentrations were used: CB1, SR 141716A (100 nM); CB2, SR 144528 (100 nM); GPR55, ML-193 (2 µM); GPR18, PSB CB5 (3 µM); and TRPV1, capsazepine (5 µM). Incubation time 72 h, resazurin test, mean ± standard error (*N* = 4 experiments). (**A**) MCF-10A, (**B**) MCF-7, (**C**) BT-474, (**D**) SK-BR-3, (**E**) BT-20, and (**F**) MDA-MB-231. *, a statistically significant difference from 2-ADFP alone, *p* ≤ 0.05, ANOVA with Holm–Sidak post-test.

By their response to the 2-ADFP treatment, the cell lines could be separated into three groups (Figure 6):

- MCF-10A: Neither of the receptor blockers used caused a substantial reduction in the substance activity.
- BT-474 and BT-20: The CB2 receptor blocker substantially decreased 2-AG activity.
- MCF-7, SK-BR-3, and MDA-MB-231: Both CB2 and TRPV1 receptor blockers substantially decreased the 2-AG activity.

In most cases, the addition of LPI shifted the signaling from CB2 towards TRPV1. These data point to the participation of these receptors in the response to 2-ADFP, while the CB1 receptor should be ruled out.

### 2.7. Signal Transduction Downstream the CB2 Receptor

At first glance, the observed participation of both TRPV1 and CB2 receptors in the cytotoxic activity of 2-ADFP seemed somewhat counterintuitive. However, the unifying activity of these receptors is the increase in the intracellular Ca^2+^ [47,48]. The increase in Ca^2+^ could lead to the activation of the CaMKII/CREB pathway, which in turn stimulates the expression of the COX-2 enzyme [49]. Once produced, it can oxidize the arachidonic moiety of 2-AG to PGE_2_, which is able to induce apoptosis [50]. To validate the participation of this pathway, we used several inhibitors to block some of the key signal transduction events: diclofenac (COX-2, 3 µM), N-acetylcysteine (NAC, removes oxidative stress linked to the PGE_2_-glycerol toxicity [50], 10 µM), Xestospongin C (inhibits Inositol trisphosphate receptor IP3R [51], 3 µM), and 666-15 (blocks activity of CREB [52], 3 µM). As the model, we used the MDA-MB-231 cell line with a known endogenous COX-2 expression, and BT-20, which does not express COX-2 [53]. The cell lines were chosen to represent two possible cases of the proposed core cell death induction stage. If COX-2 induction was not the case, then in BT-20 cells its inhibitor would not affect 2-ADFG activity in contrast to the MDA-MB-231 cells.

**Figure 7:**
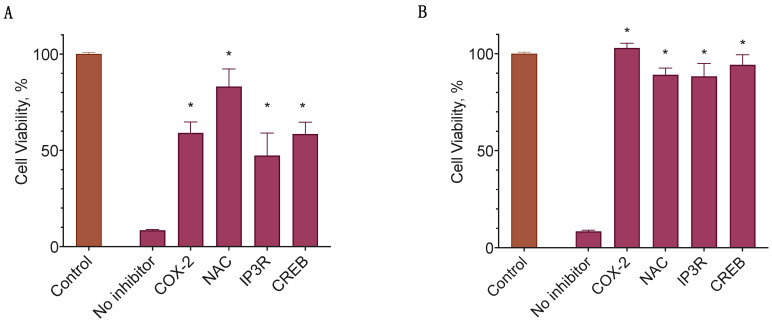
The participation of the COX-2 in the 2-ADFP cytotoxicity. (**A**) MDA-MB-231; (**B**) BT-20. Incubation time 72 h, resazurin test, mean ± standard error (*N* = 4 experiments). *, a statistically significant difference from 2-ADFP without inhibitor, *p* ≤ 0.05, ANOVA with Holm–Sidak post-test.

In both cell lines, NAC and diclofenac substantially reduced the cytotoxic effect of 2-ADFP, indicating the crucial role of the enzyme (Figure 7). Xestospongin C was also active in both cell lines; however, in the MDA-MB-231 cell line, it only reduced the cytotoxicity by about 50%. This agrees with the data on the participation of the TRPV1 receptor in the 2-ADFP signaling in this cell line.

## 3. Discussion

In this paper, we synthesized the new 2-AG analog resistant to acyl migration. The same approach was previously applied to the 2-methyl derivative of 2-AG [54]. The replacement of both hydroxyl groups by fluorine atom completely blocks the migration of arachidonoyl moiety. We developed an easy method to prepare 2-ADFP via the esterification of arachidonic acid with 1,3-difluoro propanol that substituted the glycerol moiety of 2-AG. This molecule could be easily obtained by the F-anhydride method [55]. Molecular docking experiments, together with the data of the CB2 receptor knockdown experiments, demonstrated that 2-ADFP should have a similar affinity for the CB2 receptor, but in the case of the CB1 receptor, it behaves more like an antagonist. Thus, we did not expect to observe any CB1-based effects for this compound.

Further, we studied the effect of 2-ADFP and its modulation by LPI in a panel of human breast cancer cell lines of different tumor grades. This interaction could be quite important in the cancer setting, as the endocannabinoid system is a possible target for anti-cancer therapy [56], and LPI is usually considered a pro-proliferative molecule [57]. We found that depending on the receptor set of the cell line, the 2-ADFP effect could occur via different receptor targets, and usually, it is enhanced by the LPI presence.

2-ADFP induced cell death in all six cell lines studied. The obtained EC_50_ values (89–167 µM) were relatively low compared to the typical data on the 2-AG activity from the literature [58], but were in line with our data on the low CB2 receptor expression on these cell lines [31]. On the other hand, for the DU 145 cells, which express larger quantities of CB2, the EC_50_ (1.37 µM) value was close to the ones typically observed in the literature. The addition of LPI resulted in the dose-dependent enhancement of the 2-ADFP cytotoxicity, but only for the malignant cell lines. This behavior was rather unexpected, as the typical activity of LPI is the stimulation of the proliferation [27]. On the other hand, we have already observed a similar interaction pattern for the LPI-anandamide pair [31].

Our next aim was to reveal the mechanisms of the observed cytotoxic effects. Since both 2-AG and LPI belong to the endocannabinoid system, we tried to block their activity using the selective blockers for the classical cannabinoid receptors CB1 and CB2, vanilloid receptor TRPV1, and non-classical cannabinoid receptors GPR55 and GPR18. In these experiments, only the blockers for the CB2 and TRPV1 receptors were active, and the addition of LPI shifted the effect towards less sensitivity to the CB2 receptor inhibition. The participation of CB2 and TRPV1 receptors in the 2-ADFP response seems to be logical, as both of these receptors recognize 2-AG [3,4,59]. The absence of the CB1 response contradicts the literature data on the mechanisms of the anti-proliferative effect of 2-AG [10,11,12]. However, given our data on the affinity scores of 2-ADFP towards different CB1 receptor conformations, it could probably be due to the antagonism of 2-ADFP for the CB1 receptor. The lack of response to the GPR55 and GPR18 receptor blockers in the experiments on the LPI activity could be due to the participation in this interaction of its other receptor GPR119 [60].

In the context of the breast cancer cell lines, CB2-based cytotoxicity is a rather unexpected behavior, as the main signal route for it, ceramide biosynthesis, is counterbalanced by the sphingosine-1-phosphate synthesis in this setting [19]. However, 2-AG could induce apoptosis indirectly after the oxidation to its PGE_2_ derivative [50]. We checked this possibility by the addition of the inhibitor of the involved oxidase, diclofenac. Diclofenac fully protected cells against the 2-ADFP cytotoxicity, indicating the involvement of this pathway. However, based on the literature data [53], the basal expression of COX-2 is only observed for the MDA-MB-231 cell line. On the other hand, both CB2 and TPRV1 can lead to an increase in Ca^2+^ concentration in the cells, and this event via the CaMKII/CREB activation could induce COX-2 expression. To check for the participation of this pathway, we used the IP3R blocker (CB2 activates PLC, which produces IP3, and this ligand, in turn, activates and opens the IP3R Ca^2+^ channel [49]), and CREB inhibitor 666-15. Both substances prevented the cytotoxicity of 2-ADFP, confirming the hypothesis. The calcium-dependent activation of COX-2 expression is in line with the possible activation of the GPR119 receptor by LPI, as this protein could also induce Ca^2+^ mobilization [61].

**Figure 8:**
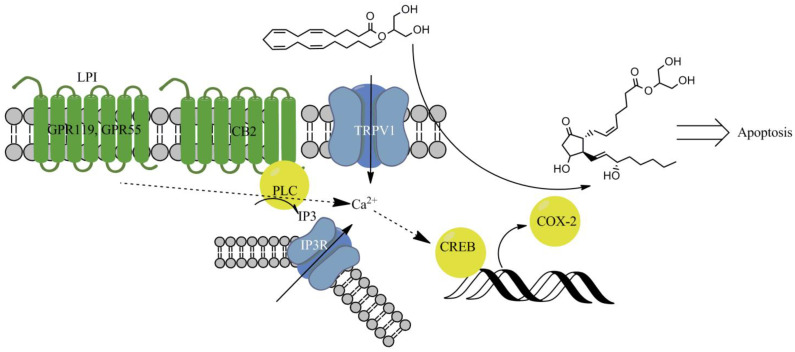
Signaling during the CB2/COX-2-dependent cell death induction by 2-AG.

The resultant sequence of events during the cell death induction by 2-AG deduced from experiments with 2-ADFP and its modulation by LPI is summarized in Figure 8.

The observed two-stage mechanism of 2-AG cytotoxicity is an interesting addition to the already known mechanisms of the activity of this compound. On one hand, it could be used as a novel principle for the rational design of anti-cancer compounds. On the other hand, the oxidation products produced by COX-2 may have pro-proliferative activity [62], and so the discovered COX-2 induction and 2-AG metabolic transformations should be taken into account during endocannabinoid-based cancer treatment development.

## 4. Materials and Methods

### 4.1. Reagents and Cell Lines

DMEM/F12 (cat. No. G4610-500ML), DMEM (cat. No. G4511-500ML), L-glutamine (cat. No. G4211-100ML), HCl, Earle’s salts solution (cat. No. G4215-500ML), Hank’s salts solution (cat. No. G4204-500ML), Versene’s solution (cat. No. G4050-100ML), antibiotic/antimycotic mixture (penicillin, streptomycin, amphotericin B, cat. No. G4015-100ML), and trypsin (cat. No. G4012-100ML) were from Servicebio, Wuhan, China. Fetal bovine serum (cat. No. FBS-11A) was from Capricorn Scientific, Wuhan, China.

The cell lines MDA-MB-231 (HTB-26), MCF-10A (CRL-10317), MCF-7 (HTB-22), BT-474 (HTB-20), BT-20 (HTB-19), SK-BR-3 (HTB-30), and DU 145 (HTB-81) were purchased from ATCC, Manassas, VA, USA.

Antibodies anti-b-actin and anti-CB2 were from Abcam, Cambridge, UK. Anti-mouse IgG antibody was from Jackson ImmunoResearch, Cambridge, UK.

SR 144028, PSB CB5, ML-184, ML-193, capsazepine, and LPI was from Tocris Bioscience, Bristol, UK. DMSO, resazurin, D-glucose, glycylglycine, acetic acid, MgSO_4,_ EGTA, dithiothreitol, acrylamide, bis-acrylamide, Triton X-100, SDS, nitro blue tetrazolium, Tris, EDTA, agarose, bicinchoninic acid, bovine serum albumin, anti-rabbit IgG antibody, diclofenac, N-acetyl cysteine, and 5-Bromo-4-chloro-3-indolyl phosphate-toluoidine were from Sigma-Aldrich, St. Louis, MO, USA. The purity of all the used reagents was 95% or more.

### 4.2. Chemical Synthesis

The conjugate of arachidonic acid and 1,3-difluoro-2-propanol (2-ADFP) was prepared by the F-anhydride method [55]. Briefly, 150 mg (0.5 mmol) of arachidonic acid was dissolved in 3 mL of MeCN and 70 μL of Py (0.83 mmol) and 70 μL (0.83 mmol) of cyanuric fluoride were added. The mixture was stirred at 20 C under argon for 60 min. Arachidonic acid fluoride was added to 200 μL (2 mmol) of 1,3-difluoro-2-propanol in 2 mL THF and 60 mg (0.5 mmol) of DMAP was added. The reaction mixture was extracted with EtOAc. The final product was purified on a Kieselgel 60 column, eluting with benzene (117.2 mg, 62% of theoretical). ^1^H-NMR spectrum (δ, ppm, J/Hz): 0.89 (3H, t, J = 6.6 Hz, H20), 1.40 (6H, m, H19, 18, 17), 1.83 (2H, m, H3), 2.17-2.21 (4H, m, H4, 16), 2.5 (2H, t, J = 7.5 Hz, H2), 2.91 (6H, m, H7, 10, 13), 4.59 (4H, dd, J = 4.4, J = 3.8, JH-F = 43.3 Hz -CH_2_- difluoropropanol), 5.23–5.33 (1H, dm, JH-F = 15.3 Hz, H difluoropropanol), 5.47 (8H, m, H5, 6, 8, 9, 11, 12).

### 4.3. Cell Culture

The cells were cultured in DMEM (cell lines MDA-MB-231, BT-474, MCF-7, and BT-20), McCoy 5A (cell line SK-BR-3), or RPMI 1640 (cell line DU 145) medium supplemented with 10% FBS, 4 mM (2 mM in the case of the DU 145 cell line) L-glutamine, 100 U/mL penicillin, 100 μg/mL streptomycin, and 2.5 μg/mL amphotericin B. The medium for the MCF-10A consisted of DMEM/F12 supplemented with 10% FBS, 4 mM L-glutamine, 100 U/mL penicillin, 100 μg/mL streptomycin, 2.5 μg/mL amphotericin B, 20 ng/mL EGF, 0.5 µg/mL hydrocortisone, 10 μg/mL insulin, and 100 nM (−)-isoproterenol [63]. All the cells were maintained at 5% CO_2_ and 100% humidity at 37 °C. The cells were subcultured by successive treatment with Versene’s solution and trypsin solution. Mycoplasma contamination was controlled using the Jena Biosciences (Jena, Germany) kit according to the manufacturer’s instructions.

### 4.4. Cytotoxicity and Proliferation Evaluation

The cells were plated in 96-well plates in the amount of 2500 per well in a volume of 100 µL of medium and cultured for a day. After that, the substance was added at the required concentration in the range from 0.01 to 200 μM in the form of a DMSO solution in a fresh 100 μL of the medium, in which the serum was replaced with a delipidated analog; the final DMSO concentration was 0.5% or less. If receptor blockers were used, their first portion was added in 50 μL of the medium one hour before the addition of the substance and then the second portion (to ensure the constancy of concentration) with the substance; the volume of the medium in which the substance was added was 50 µL in this case. The addition scheme was, thus, as follows: cells grown in 100 µL of the medium, inhibitor added in 50 more µL of the fresh medium, incubation 1 h, and substance + inhibitor added in 50 more µL of the medium (total well volume 200 µL). This procedure was required to maintain the inhibitor concentration after the substance addition and to avoid the medium replacement. For the proliferation induction studies, the medium with the delipidated FBS was used to eliminate the influence of the natural LPI. The cells were incubated with the substance for 72 h, after which the viability was determined using the resazurin test, and the cell death using the LDH test. Each experiment was repeated at least five times.

### 4.5. RNA Isolation and cDNA Synthesis

RNA was isolated from the in vitro cultivated cells using the QIAzol Lysis Reagent (Qiagen, Hilden, Germany) as described in the manufacturer’s protocol. The RNA samples were treated with DNase I (Thermo Fisher Scientific, Waltham, MA, USA) according to the manufacturer’s instructions. The RNA concentration was measured using a Nanodrop OneC Spectrophotometer (Thermo Fisher Scientific, Waltham, MA, USA). cDNA was synthesized with an MMLV RT kit (Evrogen, Moscow, Russia) according to the manufacturer’s recommendations.

### 4.6. RqPCR

qPCR was performed on LightCycler 96 (Roche) with qPCRmix-HS SYBR reagent (Evrogen, Moscow, Russia). Cycling conditions were 95 °C for 150 s, and then 45 cycles of 95 °C for 20 s, 57 °C for 20 s and 72 °C 20 s. Primer specificity was confirmed by visualizing DNA on an agarose gel following PCR. The relative level of expression was determined by the 2−ΔΔCt method. Beta-2 microglobulin was used as an internal control. Each analysis was performed in triplicate. The primer sequences for PCR and qPCR were as follows: beta-2 microglobulin forward 5′-CAGCAAGGACTGGTCTTTCTAT-3′, reverse 5′-ACATGTCTCGATCCCACTTAAC-3′; COX-2 forward 5′-GTGCCTGGTCTGATGATGTATG-3′, reverse 5′- CCTGCTTGTCTGGAACAACT-3′; POL2R forward 5′-CCCAGCTCCGTTGTACATAAA-3′, reverse 5′-TCTAACAGCACAAGTGGAGAAC-3′.

### 4.7. Resazurin Test

To evaluate cell viability, the culture medium in the wells was replaced with a 0.2 mM resazurin solution in Earle’s solution with the addition of 1 g/L D-glucose and incubated for 1.5 h at 37 °C under cell culture conditions [64]. After that, the fluorescence of the solution was determined at the excitation wavelength of 550 nm and the emission wavelength of 590 nm using the Hidex Sense Beta Plus microplate reader (Hidex, Turku, Finland). The positive control was the cell culture treated with the solvent alone, and the negative control was treated with 0.9% Triton X-100.

### 4.8. Cell Death Evaluation Using the Lactate Dehydrogenase Test

To evaluate cell death, an assay for the activity of the intracellular enzyme lactate dehydrogenase released into the medium from the dead cells was used [65]. To this end, the 75 µL aliquot of the culture medium from each well was transferred to a fresh 96-well plate. After that, 10 µl of the following reagents were added to each well: 36 mg/mL of lactate in phosphate-buffered saline, pH 7.2; 2 mg/mL of iodonitrotetrazolium (INT) in the diaphorase buffer (see below); 3 mg/mL of NAD^+^ mixed with 6 U/mL diaphorase in 0.03% bovine serum albumin; and 1.2% sucrose in phosphate-buffered saline, pH 7.2. The reaction mixture was incubated for 20 min at room temperature, and the optical density of the solutions was determined at the wavelength of 490 nm using the Hidex Sense Beta Plus microplate reader (Hidex, Turku, Finland). The positive control was the cell culture treated with the solvent alone, and the negative control was treated with 0.9% Triton X-100.

### 4.9. CB2 Receptor siRNA Knockdown

To test the hypothesis about the involvement of the CB2 receptor as the 2-ADFP target, the knockdown approach of this receptor using siRNA was used. Three duplex siRNAs were used: (1) sense 5′-CCAGGUCAAGAAGGCCUUUdTdT-3′, anti-sense 5′-AAAGGCCUUCUUGACCUGGdTdT-3′; (2) sense 5′- GCUUGGAUUCCAACCCUAUdTdT-3′, anti-sense 5′-AUAGGGUUGGAAUCCAAGCdTdT-3′; (3) sense 5′-CCUGGCCAGUGUGGUCUUUdTdT-3′, anti-sense 5′-AAAGACCACACUGGCCAGGdTdT-3′ [66]. The transfection of a commercially available siRNA with a random nucleotide sequence (scrambled) was used as a control.

The cells were transfected using the RNAiMax (Thermo Fisher Scientific, Waltham, MA, USA) reagent according to the manufacturer’s recommendations. The RNA and protein expression of each of the target genes was evaluated after 72 h of incubation with siRNA.

### 4.10. Molecular Docking

Ligand structures were obtained from the PubChem database (https://pubchem.ncbi.nlm.nih.gov/, access date 1 May 2022) or prepared manually using the Avogadro 1.93.0 software and optimized using the OpenBabel 3.0.0 software (http://openbabel.org/, access date 1 May 2024) [37] using the FFE force field with Fastest descent and dE ≤ 5 × 10^−6^ threshold. Protein structures were obtained from the PDB database (https://www.rcsb.org/, access date 1 May 2024) and optimized using the Chiron service (https://dokhlab.med.psu.edu/chiron/processManager.php, access date 1 May 2024) and the Chimera 1.11 software (https://www.cgl.ucsf.edu/chimera/, access date 1 May 2024) according to [67]. Molecular docking was performed using the AutoDock Vina 1.1.2 (http://vina.scripps.edu/, access date 1 May 2024). The grid center coordinates are represented in Table 4. In all cases exhaustiveness was set to 8.

### 4.11. Statistical Analysis

Statistical evaluation was performed using the GraphPad Prism 9.3 software. ANOVA with the Holm–Sidak or Dunnett post-test was used to compare the obtained values; *p* ≤ 0.05 was considered significant.

## 5. Conclusions

In breast cancer cells, the 2-AG analog 2-ADFP induces cell death using an additional two-stage mechanism. At first, it activates CB2 or TRPV1 receptor and induces CREB-dependent COX-2 expression. After that, the accumulated COX-2 oxidizes 2-AG, and its metabolites induce cell death. The observed two-stage mechanism of 2-AG cytotoxicity is an interesting addition to the already known mechanisms of the activity of this compound. On one hand, it could be used as a novel principle for the rational design of anti-cancer compounds. On the other hand, the oxidation products produced by COX-2 may have pro-proliferative activity, and so the discovered COX-2 induction and 2-AG metabolic transformations should be taken into account during endocannabinoid-based cancer treatment development.

## Figures and Tables

**Table 1:** Subtypes and major receptor expression patterns in the cell lines used [32,33].

Cell Line						
	MCF-10A	MCF-7	BT-474	SK-BR-3	BT-20	MDA-MB-231
Grade	Non-transformed	Luminal A	Luminal B	HER2	Basal	Basal
Metastasis	-	Non-invasive [34]	Low [35]	Very low [35]	Low	++
ER	-	+	+	-	-	-
PR	-	+	+	-	-	-
HER2	-	-	+	+	-	-

**Table 2:** AutoDock Vina affinity scores of 2-ADFP and 2-AG towards the CB1 and CB2 receptor in the molecular docking experiments. CB1: an antagonist-bound receptor PDB ID 5TGZ [36] and an inverse agonist-bound receptor 5U09 [37]; CB2: an agonist-bound receptor PDB ID 6KPC [38].

	Receptor		
	CB1 Inverse Agonist-Bound	CB1 Antagonist-Bound	CB2 Agonist-Bound
	Affinity Score		
2-ADFP	−7.757	−8.076	−8.687
2-AG	−7.685	−7.564	−8.638

**Table 3:** The effect of 2-ADFP on breast cancer cell line proliferation. Incubation time 72 h, resazurin test, mean ± standard error (N = 4 experiments).

	Cell Line					
	MCF-10A	MCF-7	BT-474	SK-BR-3	BT-20	MDA-MB-231
	EC_50_, µM, Mean (95% CI)					
2-ADFP	143.6 (140.6–146.6)	149.7 (143.3–156.3)	167.3 (156.8–177.7)	89.98 (83.34–97.1)	97.80 (110.7–112.3)	118.2 (116.0–120.7)

**Table 4:** Grid parameters of the docking experiments.

Protein	Center x	Center y	Center z	Grid x	Grid y	Grid z
CB1 5TGZ	42.5283	29.1777	321.53	23.254	21.82	15.6722
CB2 6KPC	11.2303	−0.326626	−45.9329	17.9808	20.3024	14.9769
CB2 5U09	20.7137	3.67531	−9.44048	16.4633	20.9743	15.9746

## Data Availability

The data presented in this study are available upon request from the corresponding author. The data are not publicly available due to legal issues.

## Abbreviations

2-AG	2-arachidonoyl glycerol
2-ADPG	2-arachidonoyl 1,3-difluoropropanol
LPI	lysophosphatidylinositol
INT	Iodonitrotetrazolium chloride
LDH	lactate dehydrogenase
ER	Estrogen receptor
PR	Progesterone receptor
BCIP	5-Bromo-4-chloro-3-indolyl phosphate
NBT	nitro blue tetrazolium
BCA	bicinchoninic acid
